# Characteristics of and risk factors for biliary pathogen infection in patients with acute pancreatitis

**DOI:** 10.1186/s12866-021-02332-w

**Published:** 2021-10-05

**Authors:** Shayan Chen, Jiyu Shi, Minghui Chen, Jun Ma, Zhaowei Zeng, Rui Wang, Yunfeng Cui, Xue Gao

**Affiliations:** 1 Department of Laboratory Science, Tianjin Medical University NanKai Hospital, Tianjin, 300100 China; 2 Tianjin Key Laboratory of Acute Abdomen Disease Associated Organ Injury and ITCWM Repair, Tianjin Medical University NanKai Hospital, Tianjin, 300100 China; 3 Tianjin Medical University, Tianjin, 300070 China; 4 Department of Surgery, Tianjin Medical University NanKai Hospital, 122 Sanwei Road, Nankai District, Tianjin, 300100 China; 5 Binzhou Medical University, Yantai City, 264003 Shandong China

**Keywords:** Acute pancreatitis, Biliary pathogens, Gram-negative bacteria, Multidrug-resistant bacteria, Risk factors

## Abstract

**Background:**

Infection in patients with acute pancreatitis, especially severe acute pancreatitis patients, is a common and important phenomenon, and the distributions and drug resistance profiles of bacteria causing biliary infection and related risk factors are dynamic. We conducted this study to explore the characteristics of and risk factors for bacterial infection in the biliary tract to understand antimicrobial susceptibility, promote the rational use of antibiotics, control multidrug-resistant bacterial infections and provide guidance for the treatment of acute pancreatitis caused by drug-resistant bacteria.

**Methods:**

The distribution of 132 strains of biliary pathogenic bacteria in patients with acute pancreatitis from January 2016 to December 2020 were analyzed. We assessed drug resistance in the dominant Gram-negative bacteria and studied the drug resistance profiles of multidrug-resistant bacteria by classifying Enterobacteriaceae and nonfermentative bacteria. We then retrospectively analyzed the clinical data and risk factors associated with 72 strains of Gram-negative bacilli, which were divided into multidrug-resistant bacteria (50 cases) and non-multidrug-resistant bacteria (22 cases).

**Results:**

The main bacteria were *Escherichia coli*, *Acinetobacter baumannii*, *Klebsiella pneumoniae* and *Pseudomonas aeruginosa*. Extended-spectrum beta-lactamase (ESBL)-producing *Escherichia coli* had a 66.67% detection rate. *Acinetobacter baumannii* had more than 50.00% drug resistance to carbapenems, ESBL-producing *Klebsiella pneumoniae* had 100.00% drug resistance, and *Pseudomonas aeruginosa* had 66.67% resistance to carbapenems. Multivariate logistic regression analysis suggested that the administration of third- or fourth-generation cephalosporins was an independent risk factor for Gram-negative multidrug-resistant biliary bacterial infection in acute pancreatitis patients.

**Conclusion:**

Drug resistance among biliary pathogens in acute pancreatitis patients remains high; therefore, rational antimicrobial drug use and control measures should be carried out considering associated risk factors to improve diagnosis and treatment quality in acute pancreatitis patients.

## Background

Biliary infection in acute pancreatitis (AP) patients, especially severe acute pancreatitis (SAP) patients, is common and has been associated with invasive clinical procedures, septicemia, intestinal barrier dysfunction and gut bacteria translocation [1]. It remains an important cause of death in patients with SAP [2]; therefore, for AP patients with bile infectious symptoms, bile samples are frequently used for clinical microbiological culture tests [3]. However, the bile bacterial spectrum and antibiotic resistance characteristics are constantly changing.

Gram-negative bacilli, which are some of the most common opportunistic pathogens in hospitals, have shown increasing trends towards drug resistance in recent years [4]. Gram-negative bacilli mainly include those in the Enterobacteriaceae family and nonfermentative bacteria. Nonfermentative bacteria often produce efflux pumps, induce position changes and enzyme production, develop various drug resistance mechanisms, and easily evolve into multidrug-resistant [5] organisms (MDROs) that are resistant to three or more types of clinical antibiotics used at the same time. In recent years, with the extensive use of antibiotics for the treatment of bacterial infections, MDROs have become common and important pathogens responsible for nosocomial infections, resulting in an intractable challenge for clinical diagnosis and treatment [6]; therefore, it is necessary to clarify the risk factors for MDRO infection in AP patients.

We conducted this study to understand the characteristics of pathogens causing biliary infections and related risk factors in patients with AP, guide clinical detection, promote the rational use of antibiotics, control multidrug-resistant bacterial infections, and improve the cure rate in AP patients. We analyzed the in-hospital data of AP patients from January 2016 to December 2020 to retrospectively identify the biliary pathogenic spectrum and drug resistance profiles, Gram-negative multidrug-resistant bacteria distributions and resistance rates, and associations of risk factors in the clinical characteristics of the patients. The study results are as follows.

## Results

### General characteristics of the distributions and proportions of bile pathogenic bacteria

**Fig. 1:**
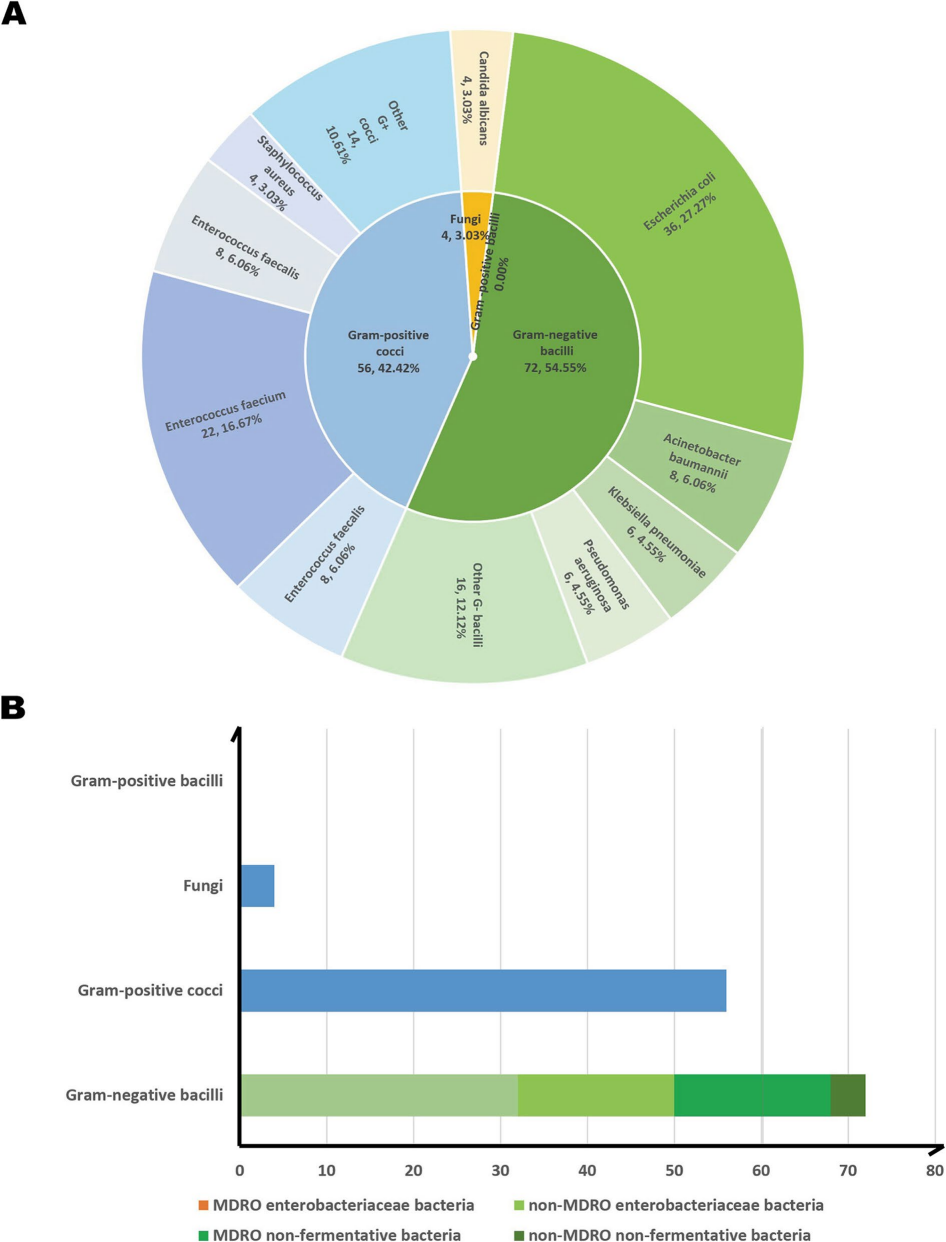
The distribution and proportion of pathogenic bacteria in AP patients. **A** The distribution and proportion of pathogenic bacteria in 120 cases AP patients. **B** The distribution and proportion of pathogenic bacteria in 120 cases AP patients by dividing of MDRO, *Enterobacteriaceae* and Non-fermenter bacteria

A total of 120 positive cases were diagnosed and 132 pathogenic bacterial strains were isolated from 560 bile samples from AP patients. Duplicate strains from the same patient were excluded. The proportion of samples with more than one pathogen was 10.00%. Seventy-two Gram-negative bacterial strains accounted for 54.55% of the isolates, 56 Gram-positive bacterial strains accounted for 42.42% of the isolates, and 4 fungi accounted for only 3.03% of the isolates. Regarding Gram-negative bacteria, here mainly referred with Gram-negative bacilli, namely the Enterobacteriaceae family and nonfermentative bacteria. The Enterobacteriaceae family included *Escherichia coli* and *Klebsiella pneumoniae,* and so on, nonfermentative bacteria covered *Acinetobacter baumannii* and *Pseudomonas aeruginosa*, and so on. Fifty strains belonging to Enterobacteriaceae accounted for 37.88%, and 22 nonfermentative bacterial strains accounted for 16.67%. According to the drug resistance profiles, the percentage of multidrug-resistant bacterial strains with at least 50.00% resistance was 37.88%; among them, 32 Enterobacteriaceae had at least 50.00% resistance, accounting for 24.24% of the total or 64.00% of the 50 Enterobacteriaceae strains isolated. Eighteen nonfermentative strains were multidrug resistant, accounting for 13.64% of the total or 81.82% of 22 strains of nonfermentative bacteria isolated. The distributions and proportions of pathogenic bacteria are shown in detail in Fig. 1A/B.

### Analysis of the drug resistance rates of Gram-negative pathogenic bacteria in the bile of AP patients

**Fig. 2:**
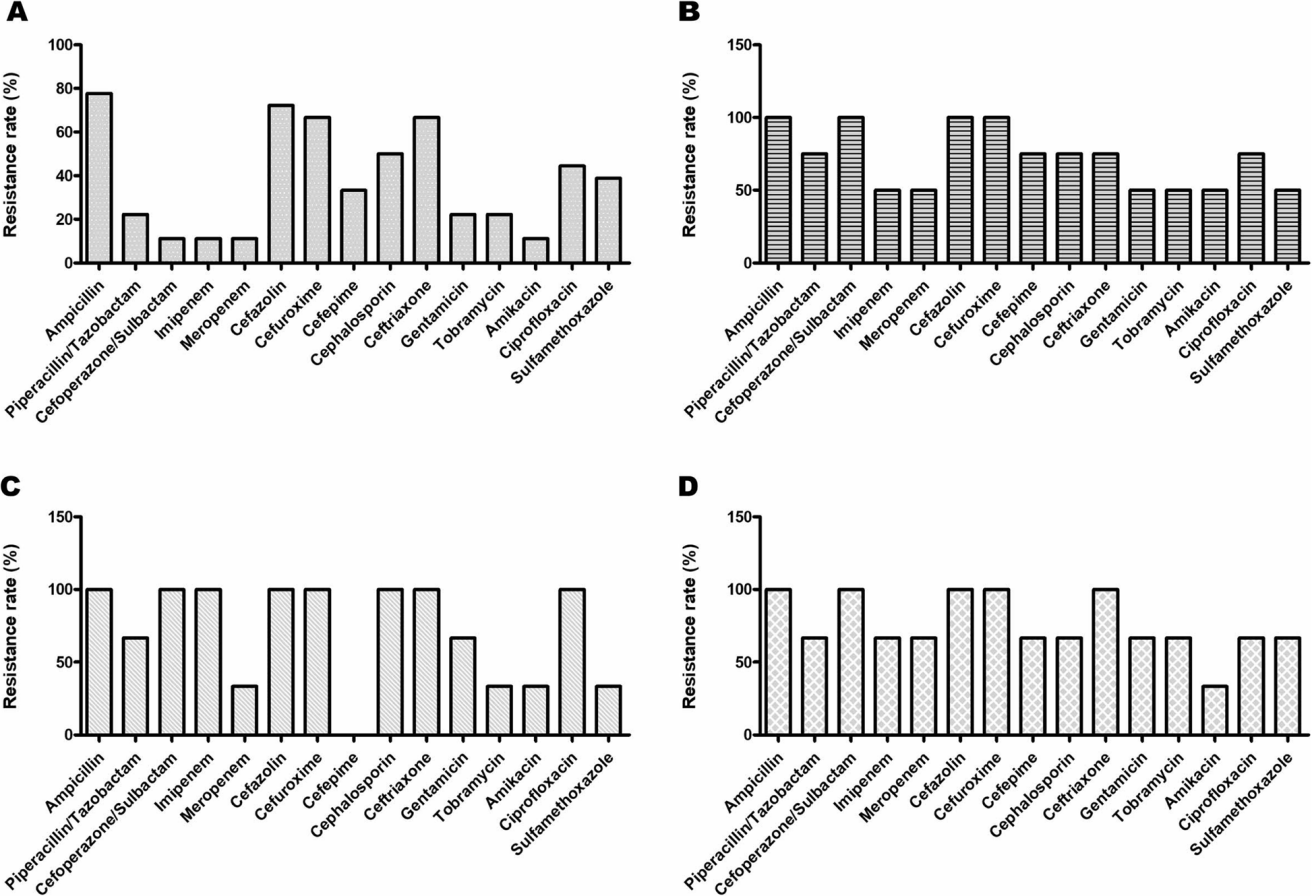
Drug resistance analysis of main Gram-negative pathogenic bacteria in AP patients. **A** Drug resistance analysis of *Escherichia coli*. **B** Drug resistance analysis of *Acinetobacter baumannii*. **C** Drug resistance analysis of *Klebsiella pneumoniae*. **D** Drug resistance analysis of *Pseudomonas aeruginosa*

In this study, the Gram-negative bacterium accounting for the largest proportion was *Escherichia coli*, followed by *Acinetobacter baumannii*, *Klebsiella pneumoniae* and *Pseudomonas aeruginosa*. The specific drug resistance rates are shown in Fig. 2.

### Resistance analysis of Gram-negative MDROs from bile samples from AP patients

**Fig. 3:**
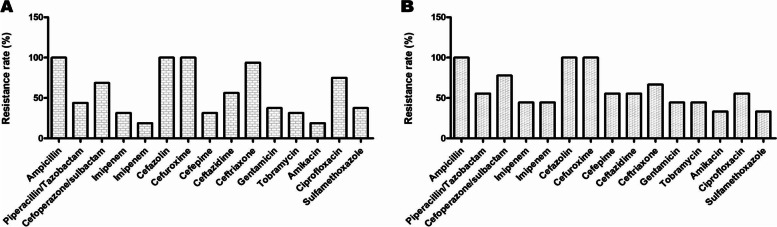
Analysis of drug resistance rates of biliary enteroderaceae and non-fermentative bacteria MDRO in AP patients. **A** Drug resistance analysis of Gram-negative enterobacteriaceae MDRO. **B** Drug resistance analysis of Gram-negative non-fermentation MDRO

Resistance of Gram-negative MDROs was further analyzed by Enterobacteriaceae and nonfermentative bacteria, and the data are shown in Fig. 3.

### Analysis of major risk factors

Analysis of major risk factors was performed according to the literature [7]. The risk factors for common MDRO infections are currently believed to be age, invasive operation, treatment with three or more antimicrobial agents, and previous multiple or long-term hospitalization. In this study, univariate analyses of 15 common potential risk factors were conducted, and the results are shown in Table 1. Multivariate logistic regression analysis was performed for 7 types of risk factors, and the results suggested that the use of third- or fourth-generation cephalosporins was an independent risk factor for Gram-negative MDRO infections in patients with AP (*P* < 0.05), as shown in Table 2.

**Table 1:** Single factor analysis of risk factors for bile Gram - negative MDRO infection in AP patients

Dangerous factor	MDRO group (*n* = 50)	Non-MDRO group (*n* = 22)	t value / Z value / χ^2^ value	*P* value
Age (Years)	60.62 ± 14.01	65.70 ± 18.22	0.8969^a^	0.3761
Hospital stays (Day)	35.65 ± 26.58	15.26 ± 14.25	−3.211^b^	<0.001
Duration of antimicrobial use (Day)	28.44 ± 25.41	12.47 ± 10.98	−4.545^c^	<0.001
Types of antimicrobial agents used (Type)	4.58 ± 3.22	2.40 ± 1.57	−4.106^d^	<0.001
Gender (Male)	30	10	0.954^e^	0.791
Use of third or fourth generation Cephalosporins	44	8	0.881^f^	<0.001
Use of Carbapenems	48	6	21.65^g^	<0.001
Invasive operation	40	6	1.050^h^	<0.001
Ventilation	6	2	1.352^i^	0.066
Operation	30	10	0.592^j^	0.541
Combined with biliary tract disease	10	8	15.472^k^	<0.001
Associated abdominal infection	20	6	0.683^l^	0.539
Combined diabetes	10	4	0.852^m^	0.594
Complicated with malignant tumor	0	0	3.246^n^	0.800
Combined with COPD or respiratory failure	4	0	4.615^o^	0.763

^a^was t value of T test, ^b-d^were Z value of Kruskal-Wallis Test, respectively, ^e-o^were χ^2^ value

**Table 2:** Multivariate logistic regression analysis of bile Gram - negative MDRO infection in AP patients

Risk factors	Partial regression coefficient	Standard error	Wald	*P* value	OR value	95% CI
Hospital stays	−0.018	0.014	0.987	0.594	1.172	0.865–1.479
Duration of antimicrobial use	0.034	0.144	1.002	0.951	1.095	0.921–1.268
Types of antimicrobial agents used	0.411	0.197	1.905	0.220	1.112	0.819–1.405
Third or fourth generation cephalosporins	0.957	0.157	2.456	0.040	2.120	0.955–3.287
Carbapenems	0.885	0.386	0.566	0.112	1.579	0.967–2.191
Invasive operation	−0.157	0.234	0.020	0.812	1.562	0.657–2.468
Combined with biliary tract disease	0.592	0.805	0.244	0.620	1.364	0.247–2.482

## Discussion

AP is a common disease of the digestive system with different clinical manifestations. Especially in SAP patients with a poor prognosis and a fatality rate as high as 30% ~ 40% due to complications, infection frequently occurs and is partly caused by pathogens that pass through biliary system. Although there are many methods to treat corresponding complications, the effect is not totally satisfactory [8]. Therefore, it is necessary to actively prevent pathogen infection in AP and avoid SAP, and important to understand the characteristics of pathogen infections and their associated risk factors [9]. At present, there are many scientific studies on AP [10], but unfortunately there are relatively few and controversial studies on pathogen infections [11], hence, it is necessary to conduct in-depth research on infectious pathogens in bile of AP patients.

In this study, the bile samples were incubated and separated by a BacT/Alert3D automated blood culture instrument [12] in accordance with international standard operation procedures for bacterial detection [13, 14]; this method can guarantee the growth and accurate identification of specific organisms, even if the samples contained multiple species [13]. According to the data obtained from bile specimens from 560 AP patients in hospitals, 120 positive cases and 132 strains of pathogenic bacteria were identified, with a positive infection rate of 21.43%, which was lower than that obtained in an analysis without stratification by sample type [15], similar to those in blood culture specimens in some hospitals in northern China without disease classification [16], and higher than those in blood culture specimens in some hospitals in central cities of China [17]. The percentage of specimens with more than one pathogen was 9.1%, which was lower than the proportion of blood culture specimens [18]. Here, the main pathogenic bacteria detected in bile were *Escherichia coli* (36 strains), *Enterococcus faecium* (22 strains), *Acinetobacter baumannii* (8 strains), *Enterococcus faecalis* (8 strains), coagulase-negative *Staphylococcus* (8 strains) and *Klebsiella pneumoniae* (6 strains). Gram-negative bacteria were mainly detected (72 cases, accounting for 54.55%), among which *Escherichia coli*, *Acinetobacter baumannii*, *Klebsiella pneumoniae* and *Pseudomonas aeruginosa* were the most abundant. It has been reported in the literature that bacteria in blood culture samples are mainly Gram-positive bacteria [19], and the second most abundant bacterial species are *Escherichia coli* and *Klebsiella pneumoniae* [17, 20]; this was not consistent with our results. Among the bile pathogens in AP patients in our study, *Escherichia coli* had a high detection rate.

Detection of pathogen resistance is an important reference for guiding clinically rational drug use; thus, we analyzed the drug resistance of the top four Gram-negative bacilli bacteria. Extended-spectrum beta-lactamase (ESBL)-producing *Escherichia coli* was 66.67% resistant to ampicillin, cefazolin and cefuroxime. The rates of cephalosporin and ceftriaxone resistance were both more than 50.00%, and the rate of ciprofloxacin resistance was greater than 40.00%, the less resistance to amikacins was detected. The drug sensitivity was lower than that reported in the literature for blood samples [21]; the reasons for this require further research.

*Acinetobacter baumannii* is an important pathogen that causes nosocomial infections in China and worldwide [22]. It is intrinsically resistant to ampicillin, amoxicillin, amoxicillin/clavulanic acid, aztreonam, ertapenem, first-generation cephalosporins, second-generation cephalosporins and fosfomycin. Although in vitro sensitivity experiments showed good results, the clinical application of these agents should not be carried out because of antimicrobial therapy failure [23]. Bacterial infection and drug resistance rates have shown increasing trends. Our study showed that *Acinetobacter baumannii* had high drug resistance and a tendency towards multidrug resistance, similar to the results of previous literature reports [18, 23]. In addition, the resistance rate for hydrocarbon enzyme alkenes was more than 50.00%, which was slightly higher than that in hospital data reported in China [24] and lower than that in some hospital reports from China [23]. The conflicting results may be due to the clinically widespread use of these drugs, the time of administration, the small number of specimens collected in this study, or other factors, such as regional differences, the specific reasons remain to be further explored. However, the results indicate that drug resistance in *Acinetobacter baumannii* is becoming increasingly serious. Since conventional disinfection methods inhibit but do not kill *Acinetobacter baumannii*, patients with weak immunity or trauma may be more susceptible to infection caused by bacteria present on the hands of clinical staff or on medical devices that are not thoroughly sterilized [25]. Therefore, the prevention and treatment of *Acinetobacter baumannii* infection should not be disregarded, and secondary infections should be closely monitored during hospital treatment and care, especially in AP patients.

The detection rate of ESBL-producing *Klebsiella pneumoniae* was 100.00%, similar to the results of other reports [26]. Resistance to cefuroxime, cefazolin, ceftazidime, ceftriaxone, ciprofloxacin, cefoperazone/sulbactam and imipenem reached 100.00%. Slight sensitivity to amikacin was detected, and the resistance was higher than that in bacteria isolated from the Intensive care unit (ICU) [27]. Similarly, *Pseudomonas aeruginosa* had 66.67% resistance to carbapenems, while it was only mildly sensitive to amikacin. The high drug resistance rate in both of these pathogens may be related to the statistical deviation caused by the number of patients enrolled, or it may be that drug resistance situation among pathogenic bacteria in the bile of AP patients is serious.

With the extensive use of antimicrobial agents in recent years, MDROs have become important pathogens of nosocomial infections. MDROs belonging to Enterobacteriaceae and nonfermentative MDROs were analyzed separately. MDROs are resistant to ampicillin, first-generation cephalosporins and second-generation cephalosporins. Moreover, the sensitivity trends of ceftazidime, piperacillin/tazobactam, cefoperazone/sulbactam, gentamicin and tobramycin were similar. However, resistance to both ceftriaxone and ciprofloxacin was higher than that to ciprofloxacin. In contrast, resistance to aminoglycosides, cefepime and carbapenems were higher. It was concluded that the drug resistance patterns of MDROs belonging to Enterobacteriaceae and nonfermentative MDROs were very different, which is not conducive to clinical anti-infection treatment in AP patients.

Analysis of resistance of MDROs and risk factors for Gram-negative MDRO infection can help identify relevant risk factors and prevent infection [28]. Our results showed that seven factors, such as the length of hospital stay, time, antibacterial drug use, antimicrobial type, use of third- or fourth-generation cephalosporins, use of penicillium carbon alkene antimicrobial agents, invasive operations, and complication of biliary tract disease, are risk factors for Gram-negative MDRO infection. Multivariable logistic regression analysis of these factors indicated that the use of third- or fourth-generation cephalosporins in AP patients with the presence of biliary Gram-negative MDROs was an independent risk factor for infection and high drug resistance. Therefore, the rational use of third- or fourth-generation cephalosporins is an important measure in preventing MDRO infection.

## Conclusions

In summary, for AP patients, the result detected in bile mainly were Gram-negative bacteria, and the main pathogenic bacteria detected in descending order were *Escherichia coli*, *Enterococcus faecium*, *Acinetobacter baumannii*, *Enterococcus faecalis*, coagulase-negative *Staphylococcus* and *Klebsiella pneumoniae*. Moreover the use of third- or fourth-generation cephalosporins of biliary Gram-negative MDROs was an independent risk factor for infection and high drug resistance. Therefore hospitals should focus on strengthening the management, prevention and control of MDRO infections.

## Methods

### Study subjects

The study recruited 560 patients hospitalized from January 2016 to December 2020. There were 304 male and 256 female patients. All the subjects were aged from 19 to 80 years, with an average age of 57.5 ± 25 years, and were firstly diagnosed with AP. Among them, 8 patients (1.43%) had severe AP as the single diagnosis, 186 patients (33.21%) of moderately severe AP had a secondary diagnosis of biliary disease, 82 patients (14.64%) of moderately severe AP had a secondary diagnosis of abdominal infection, and the remaining 284 patients (50.71%) had only AP.

The clinical specimens were collected according to the revised 2012 Atlanta Classification [29]. According to the revised 2012 Atlanta Classification, the diagnosis of AP requires the presence of 2 of the following: abdominal pain characteristic of AP; serum amylase and/or lipase activity at least 3 times higher than the referent limit; and AP signs on abdominal computed tomography or transabdominal ultrasonography. In addition, AP can be classified into two types. In this study, AP was defined as the absence of organ failure and local complications. SAP was defined as the presence of local complications and/or transient or persistent organ failure and / or exacerbations of comorbidities. AP patients, who were identified by either the International Classification of Diseases, 10th revision, (ICD - 10) code K85 or the ICD - 9 CM code 577.0, were selected.

### Sample collection

Specimens were collected when the AP patients had clinically biliary sampling symptoms or infectious symptoms of biliary tract, and no antibiotics had been used. Bacterial culture identification was performed; 2 ~ 10 ml of bile was extracted via gallbladder or bile duct puncture and drainage.

### Sample culture and bacterial detection

The bile samples were aseptically injected into aerobic and anaerobic culture bottles, mixed well, placed in a BacT/Alert3D automated blood culture instrument, and continuously monitored for 2 days. Cultures with no positive indication at the end of the process were transferred to blood agar medium for culture and judged negative if no bacteria were detected and positive when the bacteria had proliferated and the instrument detected a yellow color at a certain time. The positive bile samples in the culture bottles were transferred to blood agar medium, Sabouraud fungi medium, McConkey agar medium and chocolate agar medium (Tianjin Jinzhang Biotechnology Development Co., Ltd. Tianjin, China.), which were then placed into 371 carbon dioxide bacteria incubators at 35 °C and 5% CO_2_ for 18 ~ 24 h. If necessary, anaerobic culture bags (1.5 l anaerobic culture bags; Qingdao Haibo Biotechnology, Shandong, China) were used for further culture and separation.

### Bacterial strain identification and drug susceptibility testing

When the bacteria were obtained, they were manually sampled with a VITEK 2 compact automatic microbial analysis system. Strain identification and drug susceptibility profiles were identified by the VITEK 2 compact automatic microbial analysis system. The results of the drug susceptibility tests were analyzed and compared with the standard interpretation of the Clinical Laboratory Standardization Institute (CLSI) 2015–2019. All testing operations were conducted in strict accordance with the national clinical testing operating procedures.

### Standard quality control

Standard control strains, namely, *Staphylococcus aureus* ATCC29213, *Escherichia coli* ATCC25922 and *Enterobacter cloacae* ATCC700323 (provided by the clinical testing center of the National Health and Family Planning Commission in China), were tested in parallel.

### Independent risk factors for death were identified with as multivariate Cox regression model

Seventy-two cases with Gram-negative bacilli isolation during hospitalization from 2016 to 2020 were divided into a multidrug-resistant bacteria group (50 cases) and a non-multidrug-resistant bacteria group (22 cases), and their clinical characteristics were retrospectively analyzed to identify potential risk factors. Independent risk factors for death were identified with as multivariate Cox regression model.

## Data Availability

The datasets analyzed during the current study are available from the first author upon reasonable request.

## Abbreviations

AP: Acute pancreatitis
SAP: Severe acute pancreatitis
MDRO: Multidrug-resistant bacteria
non-MDRO: Non-multidrug-resistant bacteria
ESBL: Extended-spectrum beta-lactamase
ICU: Intensive care unit
ICD - 9 CM: International Classification of Diseases, Clinical Modification, 9th revision
CLSI: Clinical Laboratory Standardization Institute
ORs: Odds ratios
CIs: Confidence intervals
